# High performance n-type Ag_2_Se film on nylon membrane for flexible thermoelectric power generator

**DOI:** 10.1038/s41467-019-08835-5

**Published:** 2019-02-19

**Authors:** Yufei Ding, Yang Qiu, Kefeng Cai, Qin Yao, Song Chen, Lidong Chen, Jiaqing He

**Affiliations:** 1 Key Laboratory of Advanced Civil Engineering Materials, Ministry of Education, School of Materials Science and Engineering, Tongji University, 4800 Caoan Road, 201804 Shanghai, China; 2 Physics Department, Southern University of Science and Technology, 1088 XueYuan Avenue, 518055 Shenzhen, China; 3 State Key Laboratory of High Performance Ceramics and Superfine Microstructure, Shanghai Institute of Ceramics, Chinese Academy of Science, 200050 Shanghai, China; 4 School of Materials Science and Engineering, Fujian University of Technology, 350108 Fuzhou, China

## Abstract

Researches on flexible thermoelectric materials usually focus on conducting polymers and conducting polymer-based composites; however, it is a great challenge to obtain high thermoelectric properties comparable to inorganic counterparts. Here, we report an n-type Ag_2_Se film on flexible nylon membrane with an ultrahigh power factor ~987.4 ± 104.1 μWm^−1^K^−2^ at 300 K and an excellent flexibility (93% of the original electrical conductivity retention after 1000 bending cycles around a 8-mm diameter rod). The flexibility is attributed to a synergetic effect of the nylon membrane and the Ag_2_Se film intertwined with numerous high-aspect-ratio Ag_2_Se grains. A thermoelectric prototype composed of 4-leg of the Ag_2_Se film generates a voltage and a maximum power of 18 mV and 460 nW, respectively, at a temperature difference of 30 K. This work opens opportunities of searching for high performance thermoelectric film for flexible thermoelectric devices.

## Abstract (web summary)

Although flexible thermoelectric materials based on conducting polymers are attractive for energy harvesting, their performance is inferior to their inorganic counterparts. Here, the authors present a facile method to deliver inorganic nanowire films with high power factor and flexibility.

## Introduction

Nowadays, the explosive growth of wearable devices has stimulated the development of materials which can power the devices with the energy harvesting from the human body^1^. Flexible thermoelectric (TE) materials as the promising energy-harvesting materials, which can directly convert heat into electricity or vice versa and realize the self-power for wearable devices from the temperature difference between the skin and the ambient environment, have attracted increasing attention^1–8^. The TE performance of a materials is evaluated by the dimensionless figure of merit, ZT = *α*^2^*σT*κ^−1^, where *α*, *σ*, and *κ* are the Seebeck coefficient, electrical conductivity, and thermal conductivity of the material, respectively, and *T* is the absolute temperature.

Until now, the research on flexible TE materials mainly focuses on conducting polymers (CPs) and CP-based composite materials^9–12^; although much progress has been made^4,13–16^, their ZT values are still incompatible to those of the inorganic TE materials. Moreover, the CP-related materials are mainly p-type while their n-type counterpart is still lacking. For the completion of a high efficiency flexible TE module, high performance of n-type materials are very desirable.

Besides the CPs, insulating polymers are also employed for forming TE composites with inorganic TE materials. For example, most recently, Hou et al.^17^ prepared p-type Bi_0.5_Sb_1.5_Te_3_ and epoxy resin composite thick film by hot-pressing (623 K, 4 MPa) and the film shows a high power factor (PF = *α*^2^*σ*) of 840 μW m^−1^ K^−2^. Recently, flexible substrate, such as polyimide, fiber or paper, has been used to support inorganic materials for preparing high performance and flexible TE materials^17–22^. For instance, Gao et al.^21^ reported a glass-fiber-aided cold-pressing method for flexible n-type Ag_2_Te films on copy paper and the power factor value was up to 85 μW m^−1^ K^−2^ at 300 K. Choi et al.^22^ prepared n-type HgSe nanocrystal thin film by spin-coating HgSe nanocrystal solution on a plastic substrate and the film showing a maximum power factor of 550 μW m^−1^ K^−2^ at 300 K. Jin et al.^23^ deposited Bi_2_Te_3_ thick film on a cellulose fibers paper via an unbalanced magnetron sputtering technique and the composite film exhibits good flexibility and a power factor of ~250 μW m^−1^ K^−2^ at room temperature.

β-Ag_2_Se is a narrow band semiconductor with an energy gap *E*_g_ = 0.07 eV at 0 K and it transforms into a cubic superionic conductor (α-Ag_2_Se) around 407 K. β-Ag_2_Se, which exhibits high electrical conductivity and low thermal conductivity, has the great potential for n-type TE material near room temperature. Several groups have reported the TE performance of Ag_2_Se^24–26^. For instance, Ferhat et al.^27^ prepared β-Ag_2_Se bulks by a direct-reaction of the element Ag and Se in evacuated quartz tubes at 1273 K and the maximum power factor of the material was about 3500 μW m^−1^ K^−2^_,_ which is similar to that of the state-of-art material at room temperature. Most recently, Perez-Taborda et al.^28^ deposited Ag_2_Se films on glass substrates via pulsed hybrid reactive magnetron sputtering and the films showing a high power factor ~2440 μWm^−1^ K^−2^ at room temperature. Nevertheless, the films are with relatively high cost since an expensive facility is used.

Although Ag_2_Se materials with excellent TE performance at room temperature have been reported, they are all non-flexible. In this work, we developed a facile strategy to prepare n-type flexible Ag_2_Se film on a nylon membrane. The Ag_2_Se film showed a very high power factor of 987.4 μWm^−1^K^−2^ at 300 K, which is one of the best values reported for flexible n-type materials and even comparable to that of some high-ZT inorganic bulk materials at high temperatures, such as SnSe (~900 μWm^−1^K^−2^ at 773 K)^29^ and Cu_2−x_Se (1200 μWm^−1^K^−2^ at 1000 K)^30^.

## Results

### Characterization of Ag_2_Se film

**Fig. 1:**
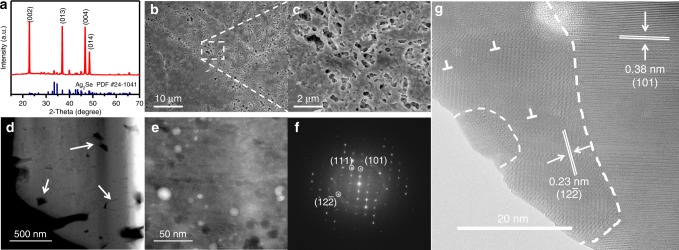
Characterization of the Ag_2_Se film. **a** XRD pattern of the Ag_2_Se film. **b** Low magnification surface FESEM image of the Ag_2_Se film. **c** High magnification surface FESEM image of the Ag_2_Se film. **d** Overview HAADF-STEM image. **e** Typical STEM image. **f** FFT image corresponding to **e**. **g** Typical HRSTEM image

XRD analysis of the film reveals that all the XRD peaks (Fig. 1a) can be indexed to β-Ag_2_Se phase (JCPDS No. 24-1041). The XRD peaks for the Ag_2_Se film are stronger than those for the Ag_2_Se nanowires (NWs) (Supplementary Fig. 3a), and especially the (002) and (004) plane peaks become particularly strong, indicating increase of crystallinity and a large number of the Ag_2_Se grains preferentially grown along the (*00l*) plane^31^. The thickness of the Ag_2_Se film is about 10 μm (Supplementary Fig. 5). After hot pressing, the Ag_2_Se NWs with diameter of ~65 nm and length of a few micrometers are sintered into a network-like film with numerous submicron pores (Fig. 1b, c).

To investigate more detailed microstructure of the film, transmission electron microscope (TEM) sample was prepared by FIB and studied by high-angle annular dark field scanning TEM (HAADF-STEM). Figure 1d shows an overview HAADF-STEM image. Although pores ranging from dozens of nanometers to several-hundred nanometers and microgaps indicated by arrows can be observed, generally interior of the film is also dense. Figure 1e shows a typical STEM image. The white dots in Fig. 1e are Pt introduced during the TEM sample preparation. Figure 1f is a FFT pattern corresponding to Fig. 1e. Figure 1g is a typical high-resolution STEM (HRSTEM) image, in which the dot-lines shows grain boundaries, showing well sintered of the Ag_2_Se nanograins with different orientations and defects (edge dislocations and stacking faults). XPS analyses (see Supplementary Fig. 6) indicate that the film is stable and no oxidation occurred during the processing.

### TE properties of the Ag_2_Se film

**Fig. 2:**
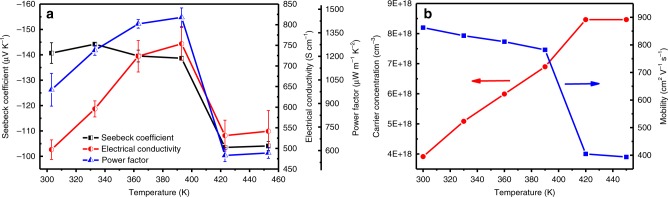
In-plane TE properties of the film. **a** Temperature dependence of Seebeck coefficient, electrical conductivity and power factor for the Ag_2_Se film (Each point shows the standard deviations from two independent measurements). **b** Temperature dependence of carrier concentration and mobility for the Ag_2_Se film

Figure 2a, b exhibit the TE properties and Hall measurement results of the Ag_2_Se film from 300 to 453 K, respectively. The Seebeck coefficient of the film at 300 K is about −140.7 μV K^−1^, indicating n-type conduction. As temperature increases, the absolute Seebeck coefficient shows a decrease tendency and it decreases rapidly when the temperature increases from 393 to 423 K. The electrical conductivity of the film is ~497 S cm^−1^ at 300 K, and it increases with the temperature increasing from 300 to 393 K and also decreases rapidly when the temperature decreases from 393 to 423 K. The temperature dependence of the Seebeck coefficient and electrical conductivity of the film can be understood from the Hall measurement results (see Fig. 2b), since the Seebeck coefficient is inversely proportional to the power of 2/3 of the carrier concentration^9^ and the electrical conductivity, carrier concentration (*n*) and carrier mobility (*μ*) have the relation: *σ* = *neμ*.

However, the change tendency above 393 K of the electrical conductivity of the film is not as that for the Ag_2_Se film reported in ref. ^28^, which is mainly because the carrier mobility of our film drops substantially (from ~800 to 400 cm^2^ V^−1^ s^−1^) while that of the latter only drops from ~600 to 500 cm^2^ V^−1^ s^−1^. The temperature dependence of carrier concentration and carrier mobility of the film is similar to that of the bulk Ag_2_Se reported in ref. ^24^, and it suddenly changes around 400 K which is due to the phase transformation from β-Ag_2_Se to α-Ag_2_Se around 407 K. The carrier concentration first increases with increasing temperature, which is due to thermal excitation of carriers (the concurrent decrease in the Seebeck coefficient of the film suggests that the additional carriers are holes). When the temperature is beyond the phase transition temperature (407 K), the Ag_2_Se becomes a superionic conductor, and then the carrier concentration almost keeps constant. The mobility suddenly drops significantly across the superionic transition, the reason for which is that the Ag ions in superionic Ag_2_Se move freely and tend to scatter electrons more efficiently than a static lattice^24^.

As a result, as the temperature increases, the power factor value also shows a similar change tendency to that of electrical conductivity: increases from 987.4 at 300 K to 1448.1 μWm^−1^ K^−2^ at 393 K, then rapidly decreases to 569.2 μWm^−1^ K^−2^ at ~423 K. The power factor value at 300 K is among the best n-type flexible TE materials (See Table 1). However, it is still much lower than the best value of the bulk Ag_2_Se with optimal carrier concentration reported in ref. ^27^. In order to understand the mechanism, we hot pressed a bulk sample without nylon. The bulk sample, with similar relative density and its grains without preferential growth direction (see Supplementary Fig. 8), has a power factor of ~1340 μWm^−1^ K^−2^ at room temperature (see Supplementary Fig. 9). Hence, we deduce that the main reasons for the film with lower power factor are: the film with high porosity, its grains preferentially grown along (00*l*), and its carrier concentration not optimized.

**Table 1:** Comparison of TE performance of flexible n-type TE materials at room temperature

Materials	*α* (μV K^−1^)	*σ* (S cm^−1^)	PF (μW m^−1^ K^−2^)	Ref.
Ag_2_Te/copy-paper	−100	85	85	^21^
Cu-doped Bi_2_Se_3_/PVDF	−84	146	103.2	^43^
Ni NWs/PVDF	−20.6	4700	200	^44^
Bi_2_Te_3_/Cellulose fiber	−130	148	250	^23^
C_60_/TiS_2_	−101	390	400	^40^
HgSe	−518	20	550	^22^
TiS_2_[tetrabutylammonium]_0.013_[hexylammonium]_0.019_	−150	400	904	^45^
Ag_2_Se/Nylon membrane	−140	497	987	This work

**Fig. 3:**
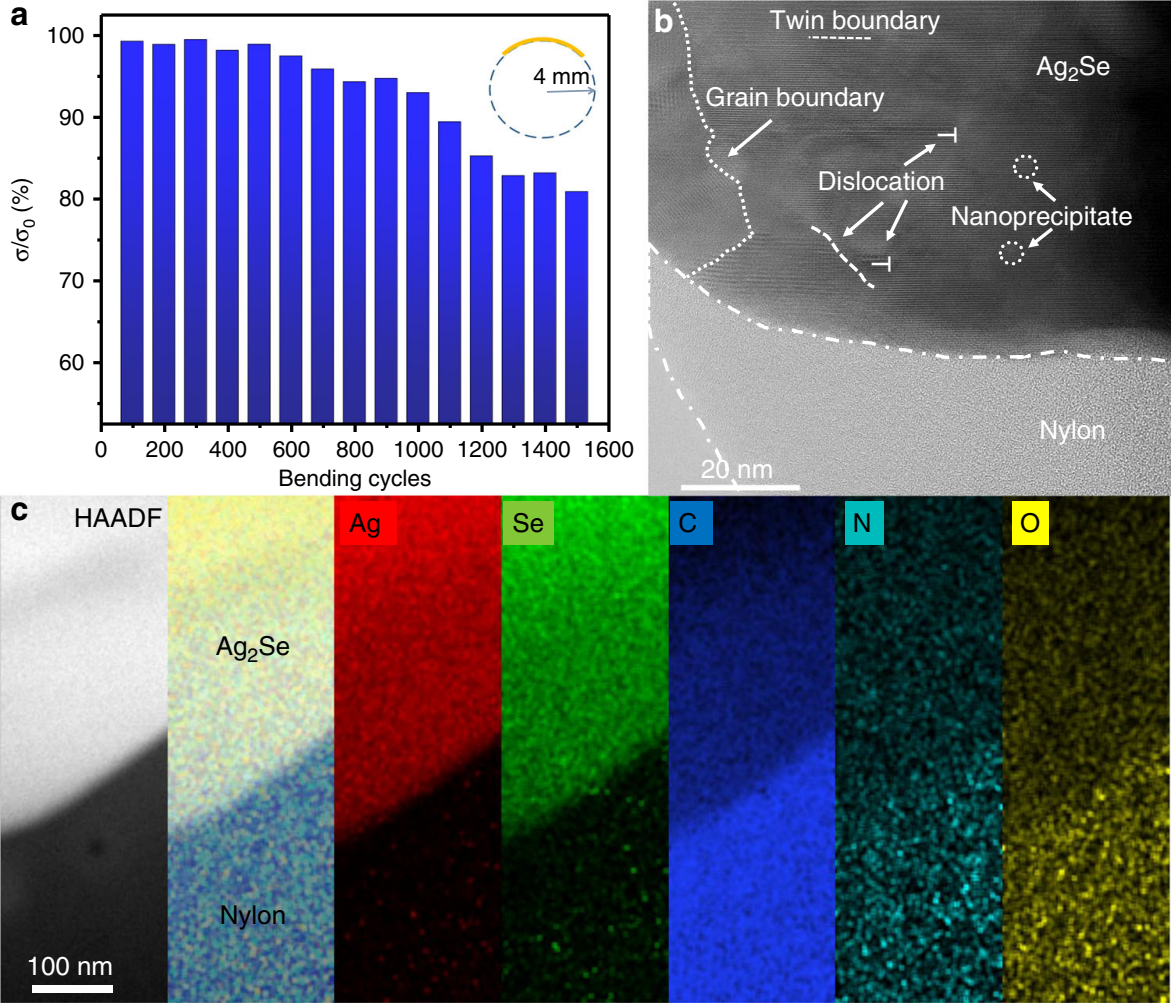
Flexibility of the film and evaluation of the heterointerface. **a** The ratio of electrical conductivity of the film before and after bending as a function of bending cycles. **b** HRSTEM image showing good combination between the Ag_2_Se film and nylon membrane. **c** From left to right: HAADF image of a heterointerface between a Ag_2_Se grain and nylon, overall corresponding EDS image and EDS image of elemental Ag, Se, C, N, and O

As it is hardly to separate the Ag_2_Se film from the nylon membrane without destroy the film, the in-plane thermal conductivity of the Ag_2_Se film cannot be provided here (the in-plane thermal conductivity of the Ag_2_Se film on nylon membrane was measured to be 0.449 Wm^−1^K^−1^, see Supplementary Table 1). However, it is deduced as follows: A *κ*_e_ (=*LσT*) of 0.268 Wm^−1^ K^−1^ is estimated for our film by using the same Lorenz number (*L* = 1.8 × 10^−8^ WΩK^−2^) as used in ref. ^28^. As our Ag_2_Se film contains pores with sizes ranging from ~20 nm to submicrometers (see Supplementary Fig. 7 and Fig. 1c), nanograins (see Fig. 1g) and a hetero-interface between the Ag_2_Se film and nylon membrane (see Fig. 3b and Supplementary Fig. 5), disorder in terms of nanocrystallite boundaries, microstructural defects (dislocations and amorphous regions), heat-carrying phonons in a wide spectrum of wavelengths can be scattered. The microstructural defects, such as dislocations (a few nm) and heterointerfaces, are effective in scattering the short wavelength phonons, and the nanocrystallite boundaries and residual nanopores (~20–200 nm) play an effective role in scattering the mid-to-high wavelength phonons^32–34^, thus the lattice contribution *κ*_l_ will be lower than that (*κ*_l_ ~ 0.21 W m^−1^ K^−1^) of the Ag_2_Se film consisting of micrograins in ref. ^28^, which suggests that the in-plane thermal conductivity (*κ*_e_ + *κ*_l_) of our film will be lower than 0.478 W m^−1^ K^−1^. Hence, the ZT value at 300 K of our film is estimated to be ~0.6.

The TE properties of a β-Ag_2_Se single crystal from 160 to 300 K are calculated using the first-principle density functional theory (see details in Supplementary Fig. 10 and Supplementary Note 5)^35^. The calculation shows that the power factor along the b-axis ((*0l0)* direction is about 2465 μW m^−1^ K^−2^ at 300 K, which is about two orders of magnitude higher than that along other two axes. Recall that the present film is preferentially grown along (*00l*) direction (see Fig. 1a) and we hereinbefore deduced that the Ag_2_Se grains grown along (*00l*) direction is not good to TE properties, which agrees with the calculation results. This implies that the TE properties of our Ag_2_Se film can be further improved via tuning the orientation of the Ag_2_Se grains, increasing the density and optimizing the carrier concentration.

### Flexibility of the Ag_2_Se film

In order to test the flexibility of the Ag_2_Se film, a bending test was applied around a rod with a diameter of 8 mm. Figure 3a demonstrates the ratio (*σ*/*σ*_0_) of the electrical conductivity with or without bending with different cycles. The electrical conductivity decreases slowly with the increasing of bending cycles. About 93 and 80% of the initial electrical conductivity are maintained after 1000 and 1500 bending cycles, respectively. Compared with the n-type Ag_2_Te films on copy paper^21^, the n-type Bi_2_Te_3_ thick film on a cellulose fibers paper^23^, and p-type PEDOT/Bi_2_Te_3_ hybrid films with monodispersed and periodic Bi_2_Te_3_ nanophase fabricated via a very complicated process^16^, our Ag_2_Se film on nylon membrane shows a better flexibility (see Supplementary Table 2).

In order to better understand the excellent flexibility of the film. We deliberately examined the details near the interface between the Ag_2_Se film and the nylon membrane. We found that the Ag_2_Se grains are well combined with the amorphous nylon membrane (Fig. 3b), indicating that there is a good bonding between them, resulted from the hot pressing, which is good for flexibility^21^. In Fig. 3c, from left to right is a HAADF-STEM image near a heterointerface, overall corresponding EDS image, elemental EDS images of Ag, Se, C, N, and O, respectively. The elements of C, N, and O are attributed to the CONH group of the nylon. It is seen from the EDS images of elemental Ag and Se that a small amount of these two elements are detected in the nylon membrane. This is because the nylon membrane is porous (pore size ~200 nm), and some tips of the Ag_2_Se nanowires may penetrate into the pores during the filtration and they bonded together during the hot pressing.

Therefore, the good flexibility of the Ag_2_Se film on nylon membrane can be explained by the following reasons. Firstly, as known to all, nylon has an intrinsic excellent flexibility. Secondly, the Ag_2_Se film is in fact a porous network intertwined with numerous Ag_2_Se nanograins sintered from large aspect ratio of Ag_2_Se nanowires; porous structure can accommodate the bending of the film^36^, hence, the film itself should have a certain flexibility. Moreover, the nylon membrane and the Ag_2_Se film have a good bonding (see Fig. 3b, c). In addition, most recently, Shi et al.^37^ reported that Ag_2_S semiconductor exhibits an extraordinary metal-like ductility with high plastic deformation strains at room temperature. As Se and S are in the same family, Ag_2_Se may have a similar ductility.

### Device performance

**Fig. 4:**
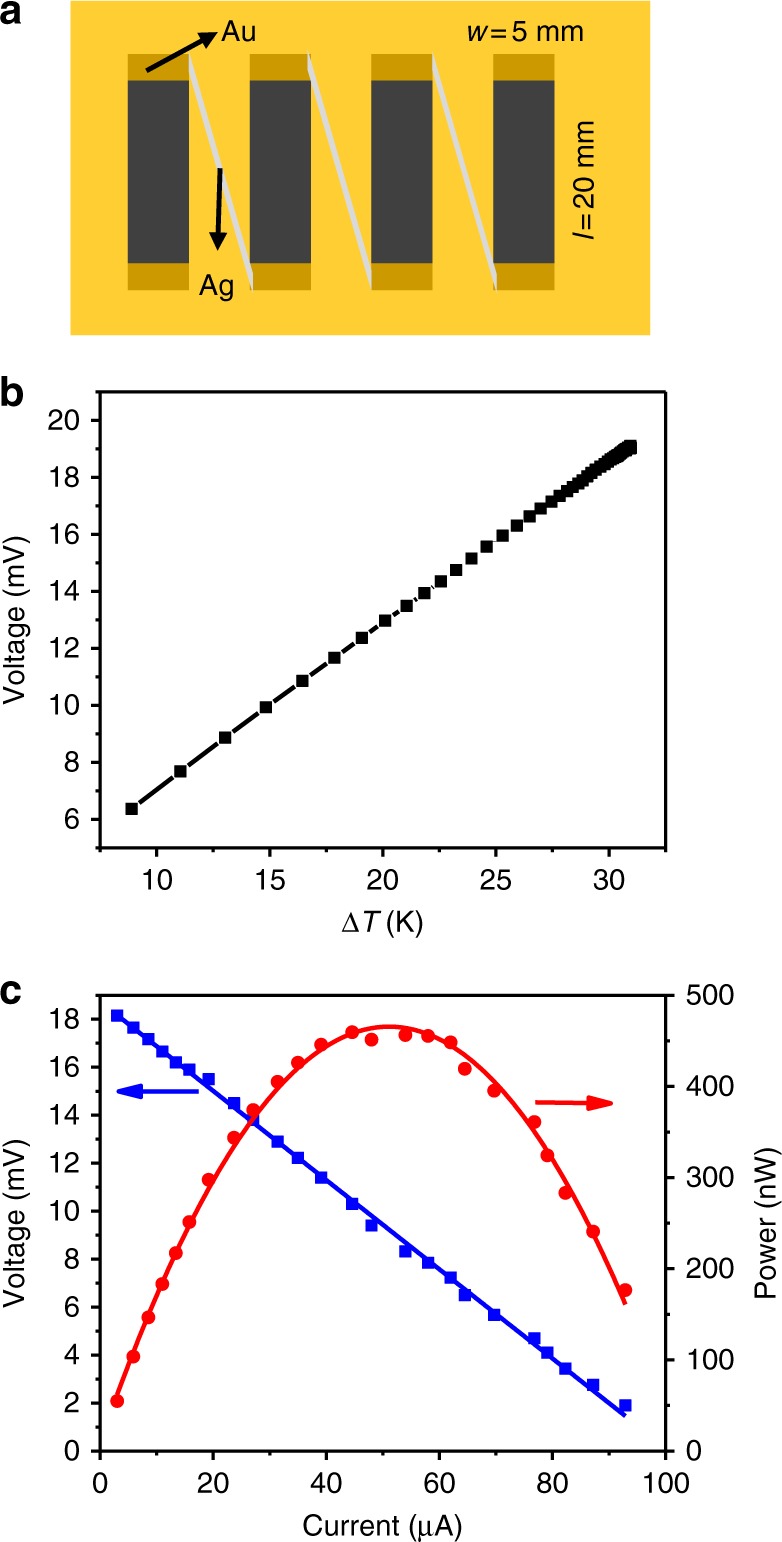
Schematic illustration and performance of the prepared device. **a** The schematic illustration of the TE device. **b** The open-circuit voltage at various temperature difference. **c** The output voltage and output power versus current at temperature difference of 30 K

As shown in Fig. 4a, the TE prototype device consists of 4 pieces of the film. Each leg is 5 mm in width and 20 mm in length. To decrease the contact resistance between the film and silver paste^38,39^, gold was first evaporated on two ends of each leg and then silver paste was painted to connect the legs in series. The open-circuit voltage and output power were measured with a homemade apparatus (see Supplementary Fig. 11 and Supplementary Note 6). Figure 4b shows the relationship between the open-circuit voltage and temperature difference. The open-circuit voltage is proportional to the temperature difference. When the temperature difference is 30 K, the measured open-circuit voltage is about 18 mV. And the calculated open-circuit voltage can be estimated by the expression of *U*_oc_ = *N*∙|*α*|∙▵*T* (*N* is the number of legs). In this case, |*α*| is about 143 μV K^−1^, thus the *U*_oc_ (=4 × 143 × 10^−3^ × 30) is about 17.2 mV, which is quite close to the measured one (the difference may be resulted from the error of the temperature (±1 K) during the measurement). The output power (*P*) is calculated by the equation as follows:

1 $${\mathrm{P}} = I^2R_{\mathrm{{load}}} = \left( {\frac{{U_{\mathrm{oc}}}}{{R_{{\mathrm{in}}} + R_{{\mathrm{load}}}}}} \right)^2R_{{\mathrm{load}}}$$

where *I* is the output current, *R*_load_ is the load resistance and *R*_in_ is the internal resistance of the device.

The curve of output voltage—current and that of output power—current are shown in Fig. 4c. The output voltage is inversely proportional to output current. At the temperature difference of 30 K, the measured maximum output power is about 460 nW. According to Eq. (1), when the load resistance (*R*_load_) equals to the internal resistance (*R*_in_) of the device, the maximum output power is obtained. And the load resistance which can be calculated from the Fig. 4c is about 250 Ω (the voltage is about 10.7 mV and the current is about 42.9 μA), matches the measurement of the internal resistance of the device. The maximum power density is about 2.3 W m^−2^, obtained from dividing the power by the cross-sectional area and the number of legs^18^, which is somewhat higher than that of reported flexible n-type devices^40–42^, confirming that the Ag_2_Se film possesses high TE performance.

## Discussion

In summary, we used a simple and low cost process compared with other published methods, i.e., first synthesis of Ag_2_Se nanowires then vacuum assisted filtration on nylon membrane and finally hot pressing at a relatively low temperature, to endow the film starting from the Ag_2_Se nanowires both good TE properties and excellent flexibility. A high power factor of 987.4 ± 104.1 μW m^−1^ K^−2^ at 300 K was obtained, which is a record value among the recently reported n-type flexible TE materials. The high power factor comes from the special Ag_2_Se film; and the excellent flexibility comes from the nylon membrane, the porous nanostructured Ag_2_Se film, and good combination of the Ag_2_Se film and nylon membrane. A prototype device with 4-leg of the film connected with silver paste was fabricated. The maximum power density of the device is about 2.3 W m^−2^ at a temperature difference of 30 K. Together with the good TE properties of the Ag_2_Se film and excellent flexibility of the nylon membrane, the Ag_2_Se film on nylon membrane has shown great promise in flexible TE modules for wearable energy harvesting. This work demonstrates an effective route to fabricate high-performance flexible TE films.

## Methods

### Synthesis

The Ag_2_Se film was prepared by a vacuum-assisted filtration of a Ag_2_Se-nanowire dispersion on a porous nylon membrane and the film on the nylon membrane was dried at 60 °C in vacuum overnight and then hot-pressed at 200 °C and 1 MPa for 30 min. See details in Supplementary Figs. 1–4 and Supplementary Note 1–2. The bulk Ag_2_Se was hot-pressed at 200 °C and 24 MPa for 30 min for comparison (Supplementary Note 3).

### Characterization

The phase composition of the Ag_2_Se film was examined by X-ray diffraction (XRD) using Cu Kα radiation (D/MAX 2550VB3+/PCII). The morphology of the film was observed by a field emission scanning electron microscope (FESEM, FEI Nova NanoSEM 450). The internal microstructure of the film was examined by double-aberration corrected transmission electron microscope (TEM, FEI Titan @300 kV in TEM and STEM mode), and the TEM sample was prepared by the Focused Ion Beam (FIB, FEI Helios600i) with the in-situ lift-out technique. To protect the sample surface before the ion milling, a Pt layer was sputtered on the full sample. The region of interest was further locally capped in the FIB with ion beam deposited carbon. The major milling was done with a 30 kV Ga ion beam while the milling progress was controlled with the scanning electron microscope. Final milling to minimize the damage layer on the specimen was performed with 5 kV followed by 2 kV Ga ion beam. X-ray photoelectron spectroscopy (XPS, ESCALAB 250Xi) was used to examine the bonding energy of the film. The pore distribution and pore volume were measured by a Brunauer-Emmett-Teller (BET) analyzer (Micromeritics, ASAP 2020).

### Measurement of TE properties and performance

The in-plane electrical conductivity and Seebeck coefficient were measured by the standard four-probe method (Sinkuriko, ZEM-3) in He atmosphere. The in-plane thermal conductivity was calculated by *κ* = *ρ*·*D*·*C*_p_, where *ρ*, *D*, and *C*_p_ are the density, thermal diffusivity, and specific heat capacity, respectively. The in-plane *D* was measured by the laser flash method (LFA467, NETZSCH), *C*_p_ was measured by the differential scanning calorimetry (DSC Q2000, TA), and the *ρ* was obtained by measuring the mass and geometrical dimensions of the film with nylon (Supplementary Note 4). The Hall coefficient was measured by the Hall measurement system (LakeShore 8404). The measurement error for *σ* and *α* is about ±5%. The thickness of the films was determined by a thickness meter (Shanghai Liu Ling Instrument Factory) combined with FESEM observation. The bending test of the film was performed using a homemade apparatus around a rod with a diameter of 8 mm.

The film was cut into strips (20 mm × 5 mm), and the strips were pasted on a polyimide substrate (the interval of two strips is ~5 mm). The two ends of each strip were coat with a layer of Au via a mask and evaporation. After that, each strip was connected in series with Ag paste as conductive connection to obtain a prototype power generator. The output voltage and output power of the device were measured by a homemade apparatus (see Supplementary Fig. 11 and Supplementary Note 6).

## Supplementary information

Supplementary Information

Peer Review File

## Data Availability

The data that support the findings of this study are available from the corresponding author upon reasonable request.
