# Supplementary material for: High performance n-type Ag2Se film on nylon membrane for flexible thermoelectric power generator
Source: Nat Commun. 2019 Feb 19;10:841. doi: 10.1038/s41467-019-08835-5 (PMC6381183; doi:10.1038/s41467-019-08835-5)
Supplement: Supplementary file 1 — Supplementary Information [file 41467_2019_8835_MOESM1_ESM.pdf]

High performance n-type Ag_2Se film on nylon membrane for flexible thermoelectric power generator

Ding et al.

Supplementary Information

High performance n-type Ag_2Se film on nylon membrane for flexible thermoelectric power generator

Yufei Ding[1], Yang Qiu[2], Kefeng Cai[1*], Qin Yao[3], Song Chen[4], Lidong Chen[3*], Jiaqing He[2*]

1. Key Laboratory of Advanced Civil Engineering Materials, Ministry of Education, School of Materials Science & Engineering, Tongji University, 4800 Caoan Road, Shanghai 201804, China

2. Physics department, Southern university of Science and Technology, 1088 XueYuan Avenue, Shenzhen 518055, China

3. State Key Laboratory of High Performance Ceramics and Superfine Microstructure, Shanghai Institute of Ceramics, Chinese Academy of Science, Shanghai, 200050, China

4. School of Materials Science and Engineering, Fujian University of Technology, Fuzhou 350108, China

*Corresponding author: kfcai@tongji.edu.cn; he.jq@sustc.edu.cn; cld@mail.sic.ac.cn

Supplementary Figures

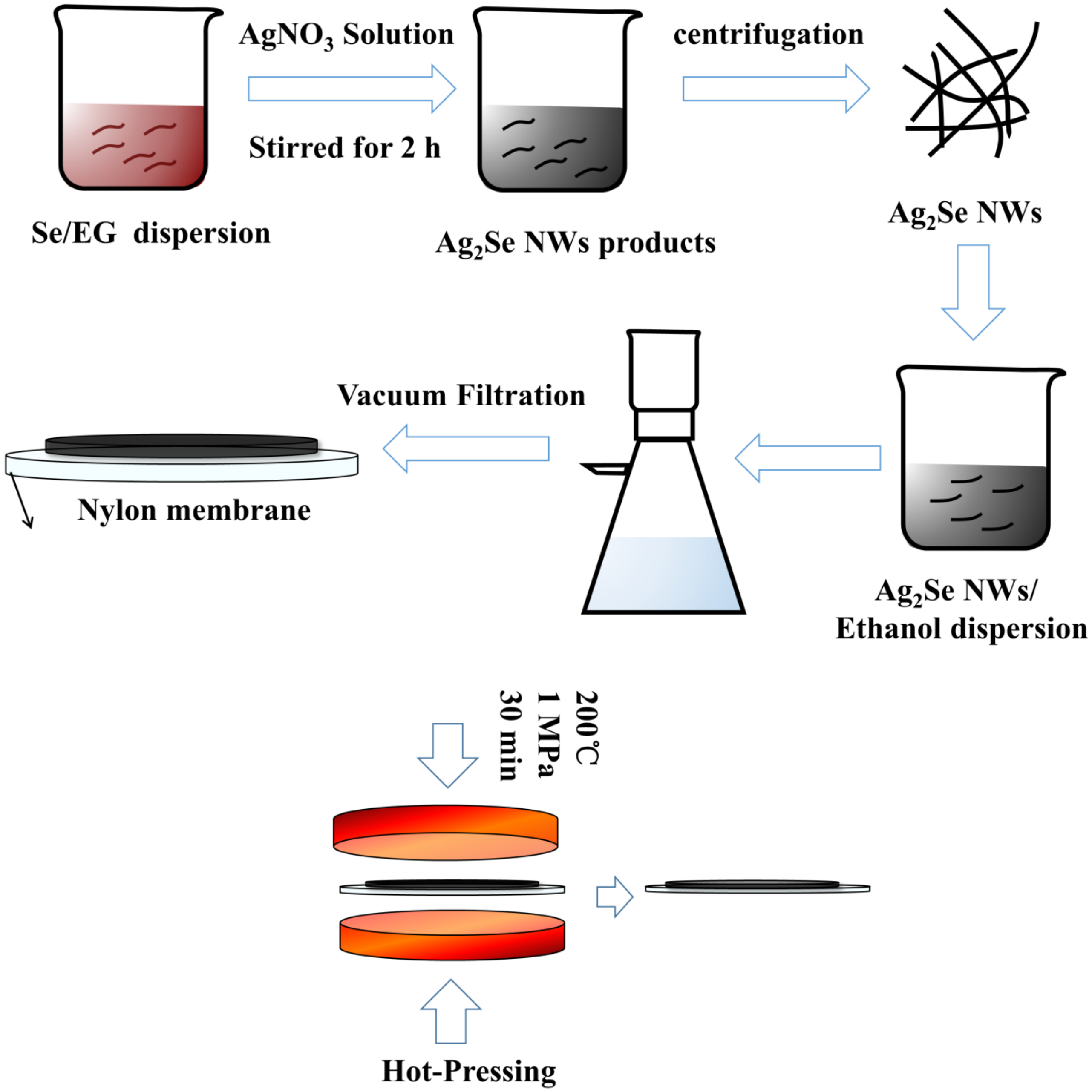

Supplementary Figure 1 Schematic demonstrating preparation of Ag_2Se film on nylon membrane

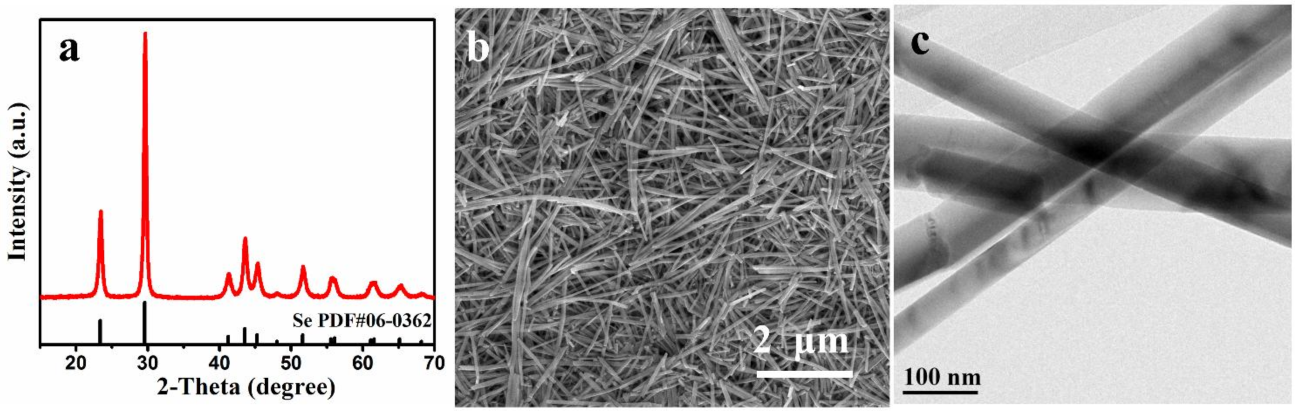

Supplementary Figure 2 Characterization of Se nanowires. **a** XRD pattern, **b** FESEM image and **c** TEM of the as-prepared Se nanowires

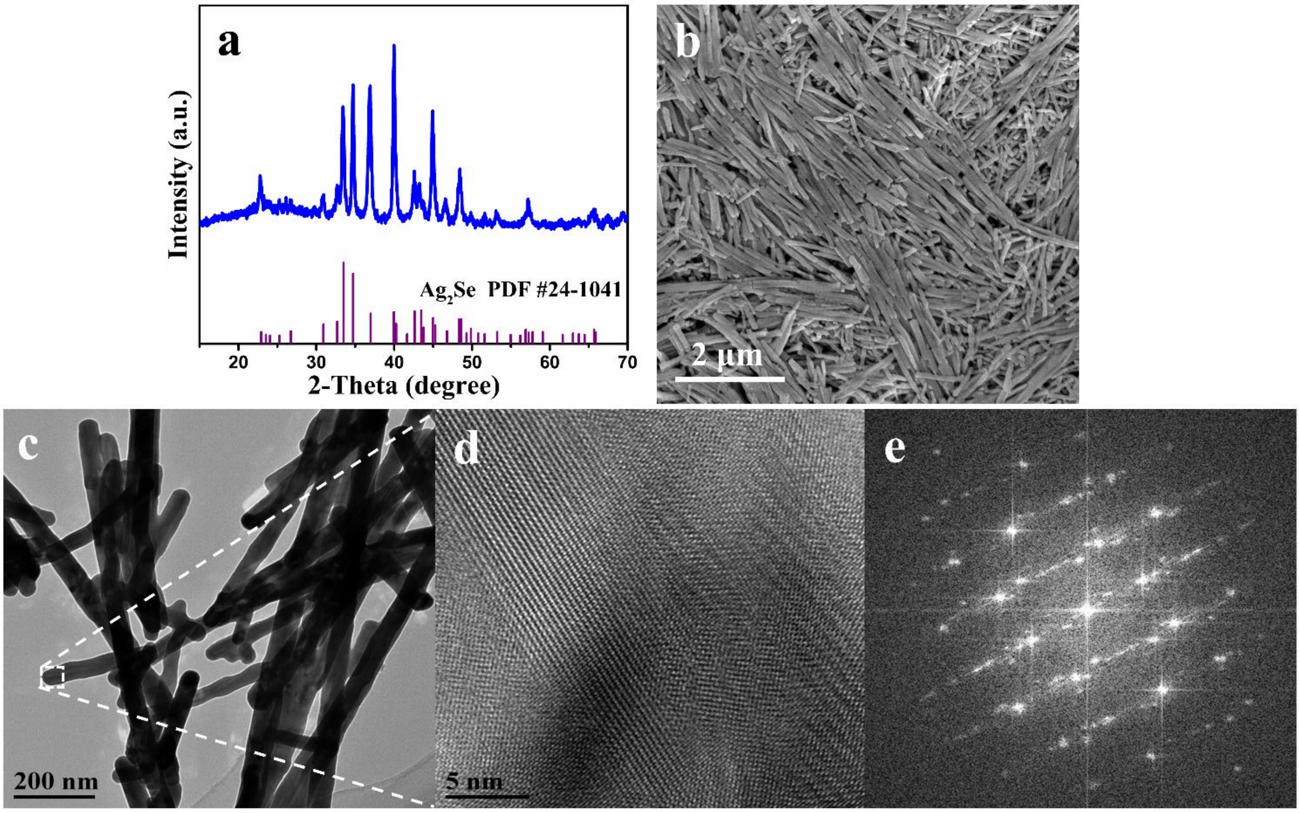

Supplementary Figure 3 Characterization of Ag_2Se nanowires. **a** XRD pattern of the Ag_2Se NWs, **b** FESEM image of the Ag_2Se NWs, **c** typical TEM image of the Ag_2Se NWs, **d** HRTEM image corresponding to the white square in **c**, **e** corresponding FFT image to **d**

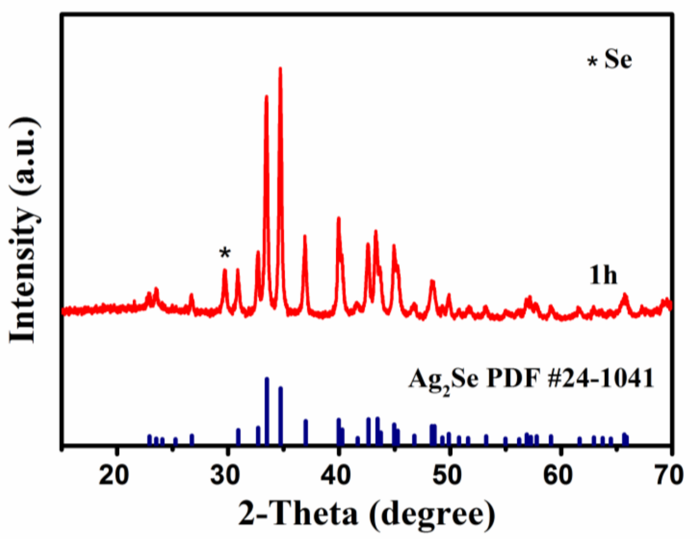

Supplementary Figure 4 XRD pattern of Ag_2Se NWs obtained from reaction for 1h

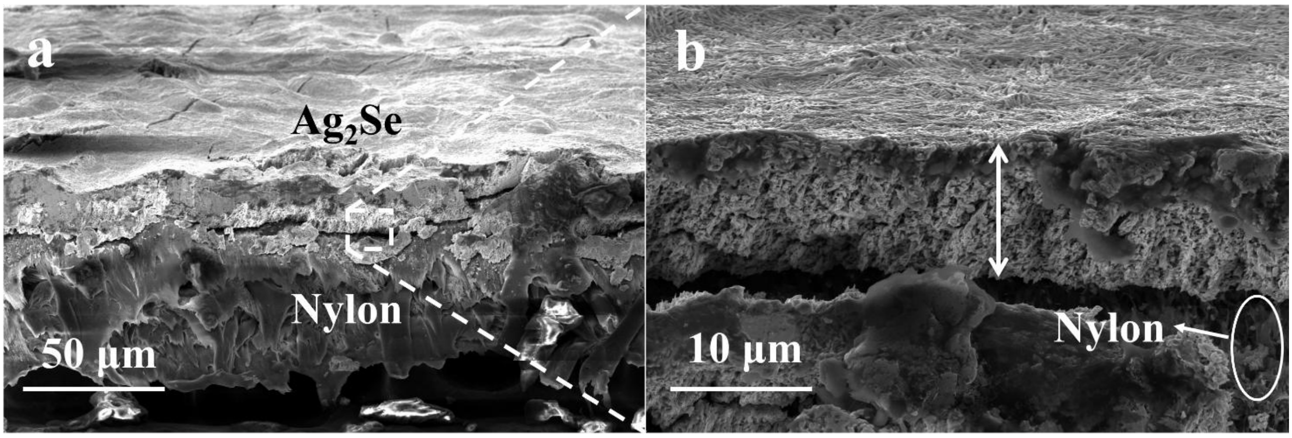

Supplementary Figure 5 Fractured surface SEM image of the hot-pressed Ag_2Se film on nylon membrane. **a** Low magnification and **b** high magnification of the fractured surface

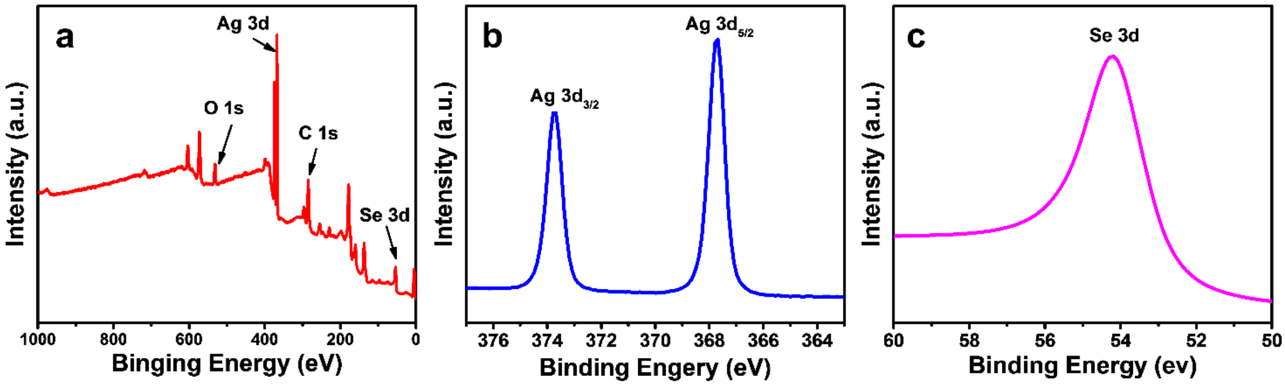

Supplementary Figure 6 The XPS spectra of the Ag_2Se film: **a** XPS survey spectrum, **b** Se 3d spectra, **c** Ag 3d spectra

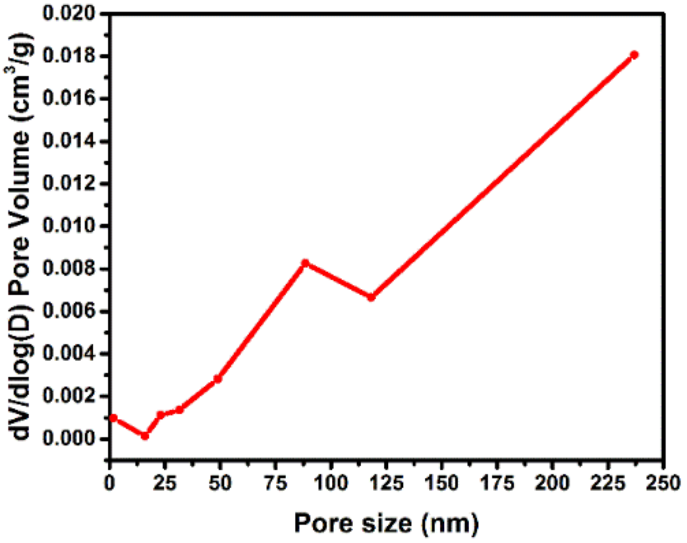

Supplementary Figure 7 Pore distribution and pore volume for the Ag_2Se film on nylon membrane

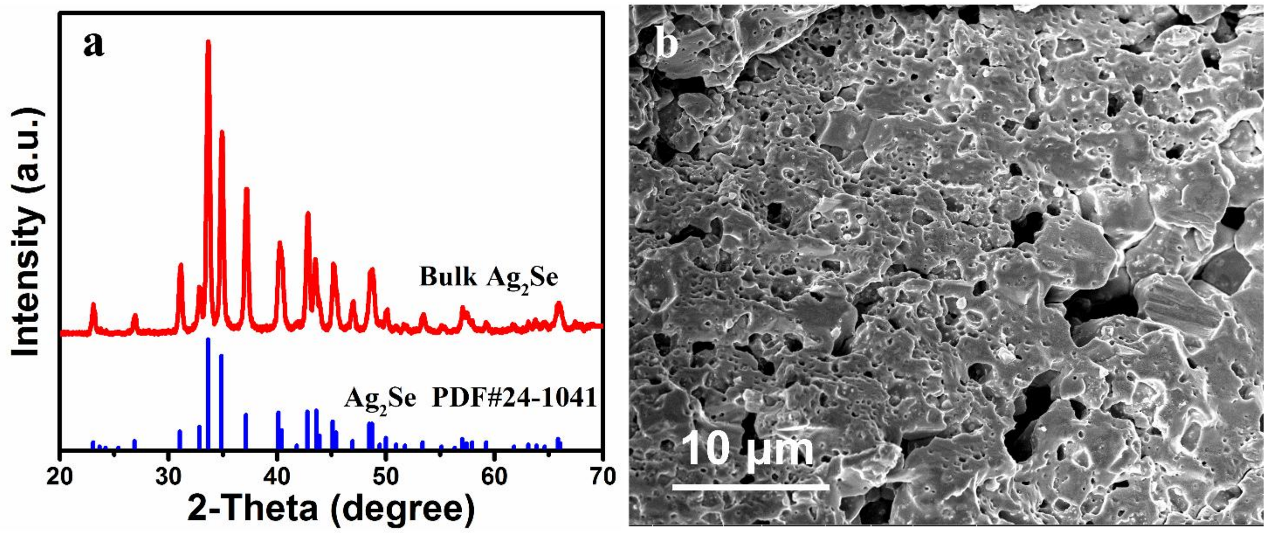

Supplementary Figure 8 Characterization of the bulk Ag_2Se. **a** The XRD pattern and **b** the cross-section FESEM image of the bulk Ag_2Se

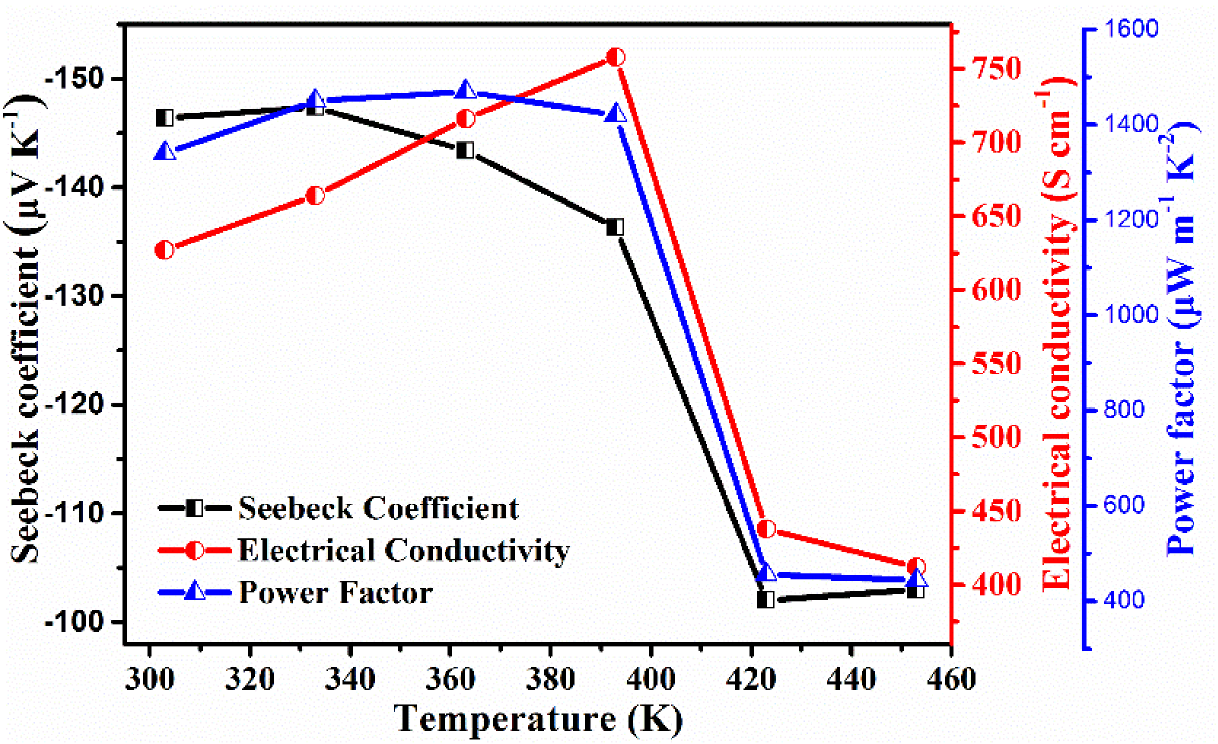

Supplementary Figure 9 Thermoelectric properties of the bulk Ag_2Se. Temperature dependence of the Seebeck coefficient, electrical conductivity and power factor for the bulk Ag_2Se

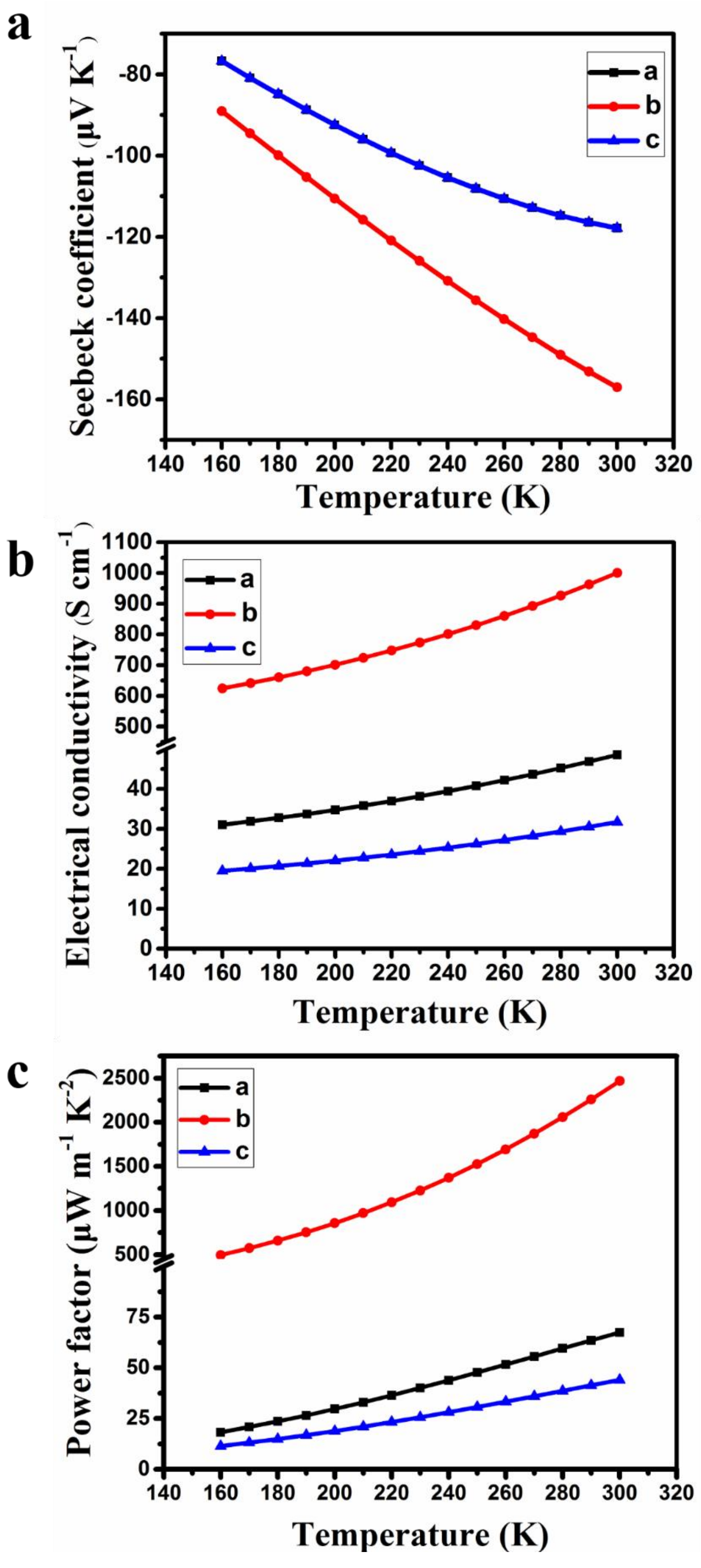

Supplementary Figure 10 Thermoelectric properties of Ag_2Se along different axes calculated by the first-principle method. The temperature dependence of **a** Seebeck coefficient, **b** electrical conductivity and **c** power factor of Ag_2Se along different axes.

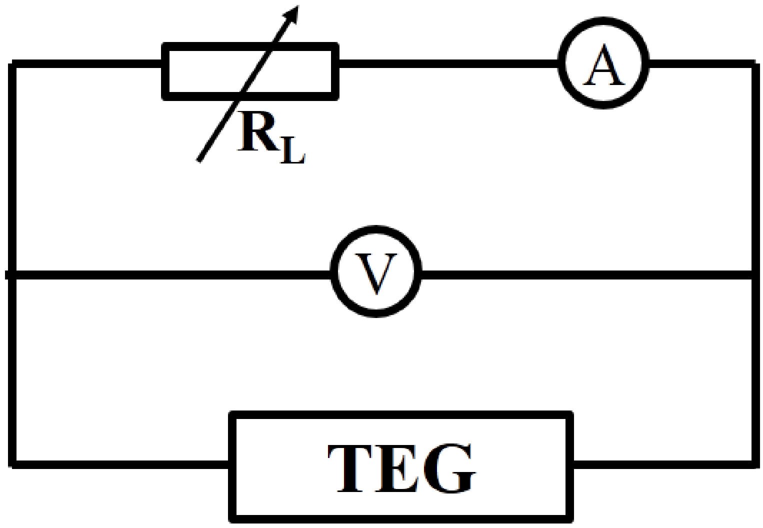

Supplementary Figure 11 Illustration of thermoelectric performance measurement.

Supplementary Tables

Supplementary Table 1 The parameters of in-plane thermal conductivity of the Ag_2Se film on nylon at room temperature

Thermal diffusivity $mm^2\ s^{-1}$	Specific heat capacity $J\ g^{-1}\ K^{-1}$	Density $g\ cm^{-3}$	Thermal conductivity $W\ m^{-1}\ K^{-1}$
0.50±0.006	0.75±0.02	1.197±0.05	0.449±0.03

Supplementary Table 2 Comparison of the flexibility of the flexible TE materials

Materials	$R/R_0 - 1$ (×100%) *	Bending radius (mm)	Bending times	Reference
Ag_2Te/copy paper	25%	5	500	[3]
Bi_2Te_3/cellulose fiber	6%	10	100	[4]
Bi_2Te_3/PEDOT	4%	3.5	100	[5]
	2%		100	
Ag_2Se/nylon membrane	7%	4	1000	This work
	25%		1500	

*all data were transferred to the increase of the resistance

Supplementary Notes

Supplementary Note 1

Synthesis of Se nanowires. The synthesis of Se nanowires was followed a method described in ref. 1[1]. In a typical procedure, 1.0 g SeO_2 and 1.0 g β-cyclodextrin were added into 200 ml distilled water in a glass beaker with stirring to form solution A. 1.0 g ascorbic acid was added into 200 ml distilled water in another glass beaker with stirring to form solution B. The solution A was dropped into the solution B with stirring. After stirring for about 4 h, the product was collected by centrifugation and washed with distilled water and absolute ethanol for several times. Then the product was stored in absolute ethanol. XRD, FESEM and TEM analyses (Supplementary Figure 2a-c) show that Se NWs were successfully synthesized. The average diameter of the Se NWs is about 60 ± 5 nm and the length is a few microns.

Supplementary Note 2

Synthesis and characterization of Ag_2Se nanowires. Ag_2Se nanowires were prepared as follows: the-as prepared Se NWs were dispersed in EG, and then a moderate $AgNO_3$ solution was dropped into the above dispersion. After stirring for 2 h, the product was centrifugated at 4000 rmp and washed with distilled water and absolute ethanol in sequence. The centrifugation and washing process was repeated for several times. XRD analysis of the Ag_2Se NWs reveals all of the XRD peaks can be indexed to β-Ag_2Se phase (JCPDS No. 24-1041), indicating that pure Ag_2Se is obtained (Supplementary Figure 3a). During the reaction, Se NWs act as templates, and EG plays the role of solvent and reductant; hence, Ag^+ cations are reduced to Ag atoms and then react with Se NWs to form Ag_2Se NWs[2]. Supplementary Figure 3b is a FESEM image of the Ag_2Se NWs, the average diameter is about 65 ± 5 nm and the length of the nanowires is several microns, which shows a large aspect ratio. Supplementary Figure 3c is a typical TEM image of the-as synthesized Ag_2Se NWs, and the average diameter is consistent with the result of the FESEM. Supplementary Figure 3a is the high-resolution TEM (HRTEM) image of the NW marked in Supplementary Figure 3c. Stacking faults, which might be induced by the template-engaged reaction, are observed, agreeing with the fast Fourier transformation (FFT) image (Supplementary Figure 3e).

When the reaction time is only for 1h, XRD analysis of the product reveals that impurity Se (JCPDS No. 06-0362) can be detected besides the Ag_2Se (Supplementary Figure 4). Therefore, we chose the Ag_2Se NWs obtained from 2 h reaction for preparation film.

Supplementary Note 3

Preparation and characterization of bulk Ag_2Se. A bulk Ag_2Se with relative density of 66% was prepared by hot pressing the nanowires in a graphite die (diameter 12.5 mm) at 200 ℃ and 24 MPa for 30 min. The XRD result and cross section FESEM image of the bulk sample are shown in Supplementary Figure 8, and the TE properties of the bulk sample are shown in Supplementary Figure 9.

Supplementary Note 4

Measurement of the in-plane thermal conductivity of the Ag_2Se film. Laser-Flash (NETZSCH LFA-467) method was used to measure the in-plane thermal diffusivity (D) of the film on nylon. To determine the thermal conductivity (κ), the density (ρ) and the specific heat capacity (Cp) at constant pressure were also measured to calculate the $\kappa = \rho C_p D$. Cp was measured by the differential scanning calorimetry (DSC Q2000, TA), and the ρ was obtained by measuring the mass and geometrical dimensions of the film on nylon. The results at room temperature are shown in Supplementary Table. 1.

Supplementary Note 5

Calculations of thermoelectric properties of the β-Ag_2Se single crystal. All the calculations were performed by means of the first-principles density functional theory (DFT), as implemented in the CASTEP code. The generalized gradient approximation (GGA) of Perdew, Burke and Ernzerh (PBE) functional was used to describe the exchange correlation interactions. Although the calculations based on PBE are known to give accurate ground state properties, PBE usually underestimates the band gap and thus the position of band edges, which may give rise to incorrect description of the electronic properties. Then, taking into account the strong Coulomb repulsion (U) of the localized d states of Ag, we chose the LDA+U approach to describe the correlation effects in transition metal silver selenide. The ultrasoft pseudopotential was used with $4d^{10}5s^1$ and $4s^2 4p^4$ as the valence electron configurations for the Ag and Se atoms. A plane-wave energy cutoff of 330 eV was used throughout all the calculations. All atomic positions and lattice constants were optimized by using the conjugate gradient method. The convergence criterion for the electronic energy was 10^{-6} eV. The structure was allowed to relax until the Hellmann-Feynman forces being less than 0.01 eV/Å. The k-point meshes of 3×3×3 and 15×15

×15, generated by the Monkhorst–Pack scheme, were used for geometry optimization and electronic structure calculations.

According to the standard Boltzmann kinetic transport theory, the electrical conductivity and the Seebeck coefficient are given by [3][6]:

$$\sigma_{\alpha\beta}(T,\mu)=\frac{1}{\Omega}\int\sigma_{\alpha\beta}(\varepsilon)\left[-\frac{\partial f_{\mu}(T,\varepsilon)}{\partial\varepsilon}\right]d\varepsilon \qquad 1$$

$$S_{\alpha\beta}=\sum_{\gamma}(\sigma^{-1})_{\alpha\gamma}v_{\gamma\beta} \qquad 2$$

where, $\sigma_{\alpha\beta}(i,k)=e^{2}\tau(i,k)v_{\alpha}(i,k)v_{\beta}(i,k)$, $\sigma(\varepsilon)=\sum\sigma(\kappa)$ and

$v_{\alpha\beta}(T,\mu)=\frac{1}{eT\Omega}\int\sigma_{\alpha\beta}(\varepsilon)(\varepsilon-\mu)\left[-\frac{\partial f_{\mu}(T,\varepsilon)}{\partial\varepsilon}\right]d\varepsilon$, in which $\varepsilon(k)$ is the band energy, $\tau(k)$ is the scattering time, μ is the chemical potential, f_{μ} is the Fermi function, $v_{\alpha}(i,k)=\partial\varepsilon(i,k)/\hbar\partial k$ is the band velocity, and Ω is the supercell volume. Based on the electric conductivity of AS1 at room temperature reported in ref.[4] the relaxation time of transport electrons was taken as constant value $\tau(k)=t_0=3.706\times10^{-15}$s before calculation.

The results are shown in Supplementary Figure 10.

Supplementary Note 6

Measurement of the thermoelectric generator performance. In the setup, one end of the TE generator is array onto a heating element controlled by an automatic temperature controlling module with a designed temperature acting as the hot side (T + ΔT), and another end is connected with a cold metal block acting as cold side (T). Two K-type thermocouples located at the two ends of the measured generator are used to measure temperature difference. Two wires are connected with two-end points of the generator using sliver paste to form a circuit. A voltmeter (Agilent 34970) connected in parallel is used to collect the output voltage of the generator, and a microammeter is used to collect the output current of the circuit. Through adjusting the value of load resistance, different output powers can be obtained.

Supplementary References

1. Li, Q. & Yam, V. W. High-yield synthesis of selenium nanowires in water at room temperature. *Chem. Commun.* **1,** 1006–1008 (2006).
2. Jeong, U. & Xia, Y. Large-scale synthesis of single-crystal CdSe nanowires through a cation-exchange route. *Chem. Phys. Lett.* **416,** 246–250 (2005).
3. Gao, J. *et al.* A novel glass- fiber-aided cold-press method for fabrication of n-type Ag_2Te nanowires. *J. Mater. Chem. A* **5,** 24740–24748 (2017).
4. Jin, Q. *et al.* Cellulose fiber-based hierarchical porous bismuth telluride for high-performance flexible and tailorable thermoelectrics. *ACS Appl. Mater. Interfaces* **10,** 1743–1751 (2018).
5. Wang, L., Zhang, Z., Liu, Y., Wang, B. & Wang, S. Exceptional thermoelectric properties of flexible organic−inorganic hybrids with monodispersed and periodic nanophase. *Nat. Commun.* **9,** 3817 (2018).
6. Singh, D. J. Electronic and thermoelectric properties of $CuCoO_2$: density functional calculations. *Phys. Review B* **76,** 85110 (2007).
